# Steroid Cell Ovarian Neoplasm, Not Otherwise Specified: A Case Report and Review of the Literature

**DOI:** 10.1155/2012/253152

**Published:** 2012-10-10

**Authors:** Paul Singh, Frank Deleon, Ralph Anderson

**Affiliations:** ^1^Department of Obstetrics and Gynecology, School of Medicine, University of Missouri Kansas City, Kansas City, MO 64108, USA; ^2^Division of Reproductive Medicine, Department of Obstetrics and Gynecology, John Peter Smith Hospital, Fort Worth, TX 76104, USA; ^3^Division of Gynecologic Oncology, Department of Obstetrics and Gynecology, John Peter Smith Hospital, Fort Worth, TX 76104, USA

## Abstract

*Background*. Steroid cell ovarian tumors, not otherwise specified, represent a unique cause of female virilization. Most commonly encountered in premenopausal women, these tumors can exist throughout a women's lifetime, from before puberty until after menopause. *Case*. Steroid cell, not otherwise specified, was diagnosed in a 70-year-old female significant for hirsutism. The patient demonstrated elevated total testosterone levels with normal gonadotropins, DHEA, and DHEA-S levels. CT imaging revealed a right ovarian mass and subsequent laparoscopic right oophorectomy yielded clinical improvement promptly. *Conclusion*. Virilization in females can occur based on ovarian or adrenal pathology. In terms of ovarian-based female virilization, many tumors exist that may induce women to demonstrate masculine features, such as pure Sertoli, pure Leydig, Sertoli-Leydig combinations, and gynandroblastomas. Each of these tumor types possesses a unique histologic pattern that allows for pathologic identification after removal. A rare source of ovarian-based female virilization is steroid cell neoplasms, not otherwise specified, that do not demonstrate these specific histologic characteristics and thus represent a diagnosis of exclusion after other causes of ovarian-based female virilization have been ruled out.

## 1. Introduction

Steroid cell, not otherwise specified, represents a subset of steroid cell ovarian stromal tumors that are histologically identified based on their absence of pathognomonic features, such as Reinke Crystals, Call-Exner bodies, and prominent nucleoli,seen in other androgen secreting ovarian tumors. These tumors typically present in premenopausal women with symptoms of androgen excess, typically manifested by virilization and extremely elevated testosterone levels. Due to the rarity of available data regarding these tumors, little is known regarding their malignant potential and metastatic behavior. We present a case of steroid cell, NOS, as the basis of virilization in a postmenopausal patient and outline its diagnosis, classification, and treatment modalities.

## 2. Case

**Figure 1:**
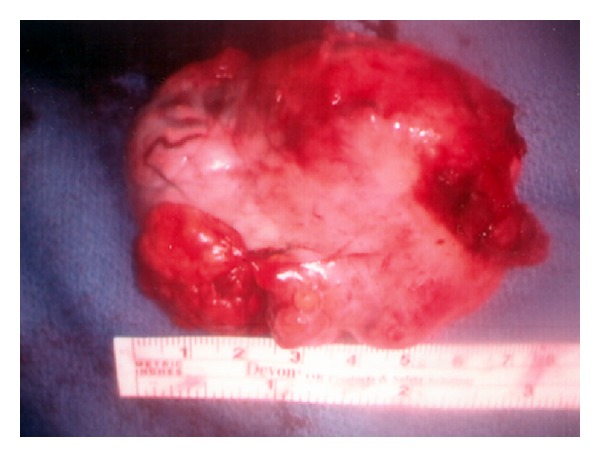
Gross specimen of right ovary with attached benign testosterone producing germ cell tumor ultimately diagnosed as steroid cell, not otherwise specified.

**Figure 2:**
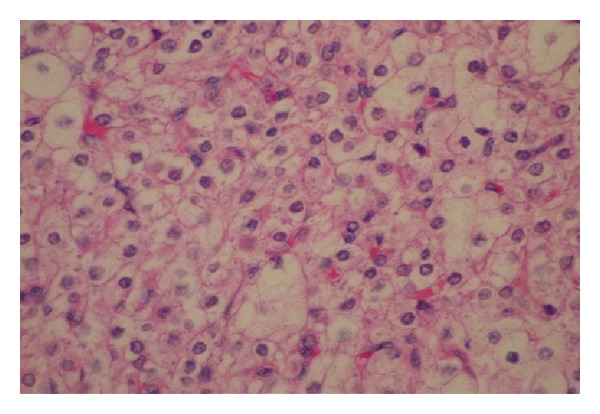
Histologic section obtained from the gross tumor. Note the eosinophilic and vacuolated appearance of the cellular structure characteristic of steroid cell, not otherwise specified.

**Figure 3:**
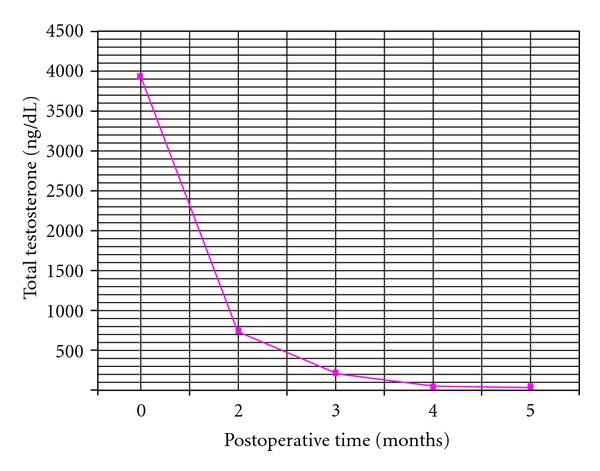
Total testosterone levels versus postoperative time. Note the abrupt reduction in the total testosterone level in the immediate postoperative period.

A 70-year-old Latin-American female with a prior medical history significant for essential hypertension, diabetes mellitus type 2, and morbid obesity presented to the reproductive endocrinology clinic with marked hirsutism over the chin and upper chest for approximately three years as well as symptoms consistent with significant male pattern balding. Laboratory workup revealed an elevated preoperative total testosterone level of 3933 ng/dL, with normal LH, FSH, DHEA, DHEA-S, 24 urine cortisol, and 17-hydroxyprogesterone levels. On pelvic examination, the patient demonstrated no clitoromegaly, a small anteverted uterus and a limited assessment of the adnexa secondary to body habitus. A CT scan of the pelvis demonstrated a lobulated uterus consistent with a pedunculated fibroid versus a right ovarian mass. Given the patient's significant comorbidities and resultant high risk for open surgery, the decision was made to proceed to the operating room for operative laparoscopy. Under direct visualization, a 5 cm × 5 cm variegated, multicystic right ovarian mass was identified (Figure 1). Biopsies of the right ovarian mass, left ovary, and peritoneum were taken and sent for frozen section which revealed no malignancy. A right oophorectomy was then performed. By the second postoperative day, the total testosterone level fell to 204 ng/dL. Final pathology of the right ovary revealed steroid cell tumor, not otherwise specified (Figure 2). After two and five months, repeat total testosterone levels were drawn in clinic, revealing normal values of 50 ng/dL and 32 ng/dL, respectively (Figure 3). The patient was then lost to follow up, and no further contact was able to be established.

## 3. Discussion

First described by Scully in 1979, steroid cell tumors represent less than 0.1% of all ovarian tumors and are grouped under the category of sex cord stromal tumors of the ovary [1]. According to the WHO, the androgen secreting steroid cell tumors are further classified as either pure sertoli, sertoli-leydig, gynandroblastomas, or steroid cell, NOS, with the most common subtype being the latter, accounting for approximately 60% of steroid cell tumors. The typical presentation of steroid cell, NOS, occurs in premenopausal women with a mean age of 43 and frequently manifests with virilization [2]. A comprehensive workup, therefore, includes the evaluation of an adrenal and ovarian source of pathology for the hyperandrogenism. Elevated testosterone levels with normal DHEA, DHEA-S, LH, FSH, and 17-OHP levels warrant abdominal imaging with ultrasound, CT, or MRI of the pelvis to look for an ovarian virilizing tumor [3]. Failure to rule out a virilizing ovarian tumor as a possible source of hyperandrogenism only results in misdiagnosis and delayed treatment. Indeed, prior case reports have demonstrated steroid cell, NOS, as the root cause of heterosexual precocious puberty incorrectly diagnosed as late onset congenital adrenal hyperplasia. Only after the workup for 17-hydroxyprogesterone levels and ACTH stimulation tests were negative and attempted management with glucocorticoid supplementation had failed, was attention turned to the ovary as the possible source of the hyperandrogenism [4].

Ultimately, histologic features obtained from permanent sectioning determine the final pathological diagnosis. Specifically, steroid cell tumors, NOS, appear as solid, well-circumscribed tumors with both eosinophilic and vacuolated polygonal cells [5]. Neither Reinke crystals, prominent nucleoli, nor Call-Exner bodies, as are seen with leydig, sertoli, and the granulosa component of gynandroblastomas, respectively, are identified in steroid cell, NOS tumors [6, 7].

Traditionally, the management for sex cord stromal cell tumors has been surgical removal of the tumor. Since steroid cell, NOS, is rarely bilateral, young patients may be treated by unilateral oophorectomy. It should be emphasized that because of a possible 5% chance of contralateral involvement, close surveillance of child bearing women who undergo unilateral oophorectomy is warranted. If future fertility is not an issue, hysterectomy, removal of the contralateral ovary, and complete surgical staging are recommended. Although most steroid cell, NOS, tumors behave in a benign fashion, malignancy has been reported in as high as 43% of cases [8]. In their work, Scully et al. described a case series of 63 steroid cell, NOS, tumors with 28.6 of them being malignant. Pathologic features that were prognostic identifiers of malignancy included two or more mitotic figures, a diameter of greater than 7 cm with necrosis or hemorrhage on the gross specimen, and grade 2 or 3 nuclear atypia. Current GOG recommendations state that for stage 2–4 disease, adjuvant chemotherapy can be implemented. An optimal adjuvant chemotherapeutic regiment has not yet been developed, with treatments by the BEP regimen and the carboplatin and paclitaxel regimen yielding equivocal results [8]. In the present case, given that our patient had multiple comorbidities and that both the frozen and permanent specimens did not demonstrate any obvious signs of malignancy, we elected to pursue a laparoscopic oophorectomy and contralateral ovarian biopsy, without staging and adjuvant chemotherapy.
